# College of Physicians and Surgeons, University of the State of N. Y.

**Published:** 1847-09

**Authors:** 


*College of Physicians and Surgeons, University of the State of N. Y.*—By reference to the advertising sheet of this Journal it will be perceived that the College of Physicians and Surgeons of N. Y., has extended its term of lectures, for the next session, to five months. This has also been done, as our readers are aware, by the University of Pennsylvania. This promptitude of action, by these time-honored institutions, in accordance with the recommendation of the National Association, cannot but serve to enhance the great respect in which they are held by the profession. We trust, and believe, that other institutions will not delay in following their example, and that the sessions of 1849–50 in all the schools, will be extended to the full period recommended by the Association.

The College of Physicians and Surgeons have, also, instituted a chair of Physiology and Pathology, and appointed, as lecturer, Alonzo Clark, M. D. Dr. Clark enjoys a high reputation as an accomplished proficient in the department of the new chair, which he has for several years taught with success of the Medical Institution of Pittsfield, Mass. His talents and reputation will add strength to the Faculty of the College of Physicians and Surgeons, and this is saying not a little, in view of the ability which that school already possessed.

